# Inverse-response Ca^2+^ indicators for optogenetic visualization of neuronal inhibition

**DOI:** 10.1038/s41598-018-30080-x

**Published:** 2018-08-06

**Authors:** Yufeng Zhao, Daniel Bushey, Yongxin Zhao, Eric R. Schreiter, D. Jed Harrison, Allan M. Wong, Robert E. Campbell

**Affiliations:** 1 Department of Chemistry, University of Alberta, Edmonton, AB T6G 2G2 Canada; 2 Howard Hughes Medical Institute, Janelia Research Campus, Ashburn, VA 20147 USA; 3 Present Address: Department of Biological Sciences, Carnegie Mellon University, Pittsburgh, PA 15213 USA

## Abstract

We have developed a series of yellow genetically encoded Ca^2+^ indicators for optical imaging (Y-GECOs) with inverted responses to Ca^2+^ and apparent dissociation constants (*K*_d_′) ranging from 25 to 2400 nM. To demonstrate the utility of this affinity series of Ca^2+^ indicators, we expressed the four highest affinity variants (*K*_d_′s = 25, 63, 121, and 190 nM) in the *Drosophila* medulla intrinsic neuron Mi1. Hyperpolarization of Mi1 by optogenetic stimulation of the laminar monopolar neuron L1 produced a decrease in intracellular Ca^2+^ in layers 8–10, and a corresponding increase in Y-GECO fluorescence. These experiments revealed that lower *K*_d_′ was associated with greater increases in fluorescence, but longer delays to reach the maximum signal change due to slower off-rate kinetics.

## Introduction

Genetically encoded fluorescent protein (FP)-based calcium ion (Ca^2+^) indicators are widely used for non-invasive monitoring of intracellular signaling dynamics in systems ranging from cultured cells to live animals^1–4^. Directed protein evolution has proven to be a highly effective strategy to develop Ca^2+^ indicators with altered fluorescence hues^5–7^ or improved performance^8–10^.

**Figure 1:**
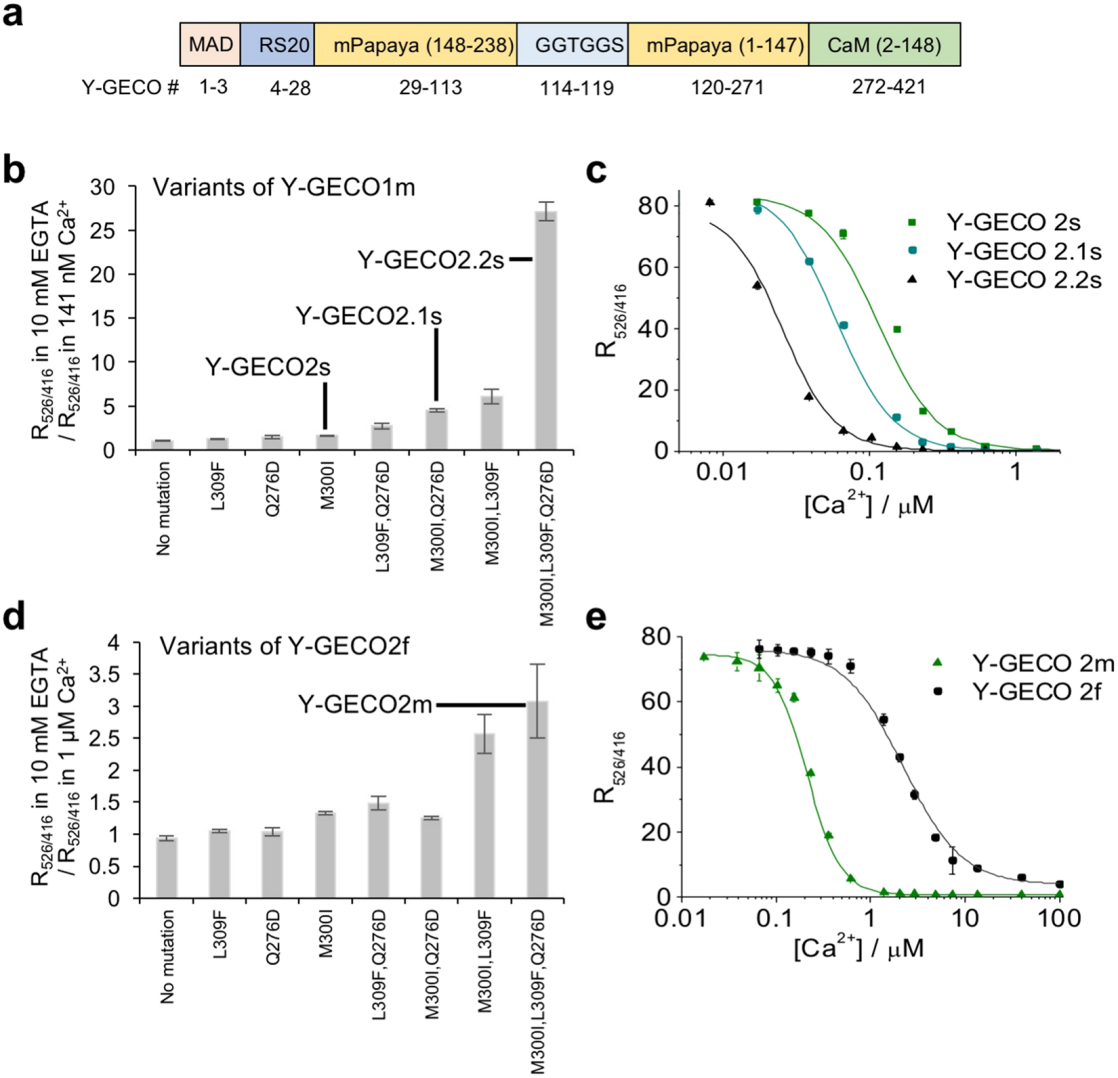
Development of new Y-GECO Ca2+ indicators. (**a**) Gene structure of Y-GECO. (**b**) Single mutation of M300I, Q276D, and L309F and combinations thereof were introduced into Y-GECO1m. The ratio of R_526/416_ in 10 mM EGTA to R_526/416_ in 141 nM Ca^2+^ was evaluated for Y-GECO1m variants. R_526/416_ is the ratio of fluorescence excited at 526 nm to fluorescence excited at 416 nm. (**c**) Ca^2+^ titrations for Y-GECO1m-derived variants. Data points were fit to Hill equation to determine *K*_d_′. (**d**) Screening of Y-GECO2f [ref.^15^] variants with mutations at the same positions as in (**b**). The ratio of R_526/416_ in 10 mM EGTA to R_526/416_ in 1.27 μM Ca^2+^ was evaluated for all variants. The variant with all 3 mutations was designated as Y-GECO2m. (**e**) Ca^2+^ titration of Y-GECO2m. Y-GECO2f data from ref.^15^. Triplicate measurements were performed and the results were averaged. Error bar represents the standard deviation of each set of measurements.

We previously introduced a first-generation microfluidic fluorescence activated cell sorter (μFACS) platform for directed evolution of FP-based Ca^2+^ indicators with higher throughput than typical manual screening of bacterial colonies^11^. This platform was applied to the development of yellow genetically encoded Ca^2+^ indicators for optical imaging (Y-GECO, Fig. 1a) based on mPapaya^12^, a monomeric variant of the *Zoanthus* sp. yellow FP^13^. Among the inverse (becoming dimmer upon binding Ca^2+^) indicators, we identified variants with both fast (Y-GECO1f) and medium (Y-GECO1, designated as Y-GECO1m in the following discussions) dissociation kinetics^11^. The Y-GECO1f indicator exhibited fast Ca^2+^-dissociation kinetics with *k*_off_ = 9.75 s^−1^, which compares favorably to Y-GECO1m (*k*_off_ = 1.40 s^−1^) and GCaMP6f^9^ (*k*_off_ = 2.32 s^−1^) measured under the same conditions. However, the improved kinetics were also associated with substantially decreased Ca^2+^ affinity (*K*_d_′ = 2.5 μM for Y-GECO1f vs. 190 nM for Y-GECO1m), and Ca^2+^-dependent fluorescence change (i.e., the change for Y-GECO1f is ~32% that of Y-GECO1m). The inverse response of the Y-GECO series of indicators is similar to that of inverse-pericam (IP)^4^ and the recently reported IP2.0 (ref.^14^).

To develop Ca^2+^ indicators tailored to specific applications in biology, we have used site-directed mutagenesis to modify the Ca^2+^ affinities and off-rate kinetics of Y-GECO variants. We demonstrate that these inverse response indicators are particularly well-suited for imaging of inhibitory neuronal signaling associated with transient decreases in intracellular Ca^2+^ concentration in neurons within the *Drosophila* visual system.

## Results

### Development of Y-GECO variants with higher affinity and slower kinetics

To expand the Y-GECO series of indicators to include variants with higher affinity for Ca^2+^, we explored the introduction of three previously reported mutations, M300I, Q276D, and L309F, in Y-GECO1m (*K*_d_′ = 190 nM). One of these mutations (M300I) represents the reversion of a mutation in the first Ca^2+^-binding site of Y-GECO that was acquired during the earlier development of Y-GECO1f^11^. The other two mutations were reported to contribute to the increased Ca^2+^ affinity of jRGECO1a^10^. Individually, all three mutations increased the affinity to Ca^2+^ (Fig. 1b). We designated Y-GECO1m M300I as Y-GECO2s (*K*_d_′ = 120 nM), Y-GECO1m Q276D M300I as Y-GECO2.1 s (*K*_d_′ = 63 nM), and Y-GECO1m Q276D M300I L309F as Y-GECO2.2s (*K*_d_′ = 25 nM) (Fig. 1c and Supplementary Tables 1–3). These variants are designated with an ‘s’ due to the slower kinetics (Supplementary Fig. 1a), which are a consequence of their higher Ca^2+^ affinities. These Y-GECO variants exhibited very similar pH dependence (Supplementary Fig. 2b–d).

### Development of Y-GECO variants with lower affinity and faster kinetics

In an effort to identify variants with fast and large fluorescent responses, we developed and used a μFACS system based on a polydimethylsiloxane (PDMS) microchip with a two-point detection system (work described in ref.^15^). This system allowed us to screen libraries of randomly mutated Y-GECO1f variants expressed in *Escherichia coli*. Use of this system led to the identification of Y-GECO2f (Supplementary Table 1) that is 26% brighter in the Ca^2+^-free state, and exhibits a greater than 300% larger Ca^2+^-dependent fluorescence decrease relative to Y-GECO1f (Supplementary Table 2) while retaining a similar spectral profile. Y-GECO2f retained a similar Ca^2+^ affinity and slightly slower *k*_off_ than Y-GECO1f (Supplementary Table 3).

While Y-GECO1f and Y-GECO2f exhibit relatively fast kinetics, they also share relatively high apparent dissociation constants of 2.5 μM and 2.2 μM, respectively. In an effort to lower the *K*_d_′ to better match the typical concentrations of Ca^2+^ in cytoplasm (~0.1 to 1 μM), we introduced the three mutations discussed earlier (M300I, Q276D, and L309F) (Fig. 1d**)**. Ultimately we found that the combination of all three mutations together gave the highest affinity, with a *K*_d_′ of 204 nM (Fig. 1e). Accordingly, Y-GECO1f Q276D M300I L309F was designated as Y-GECO2m (Supplementary Table 1). As expected for a higher affinity variant, the Ca^2+^ dissociation kinetics of Y-GECO2m had slowed and were similar to Y-GECO1m (Supplementary Fig. 1b and Supplementary Table 3). Relative to Y-GECO1m, Y-GECO2m exhibits a larger Ca^2+^-dependent fluorescence change (over 200% increase) when excited at 526 nm (Supplementary Fig. 3), due to the shift of p*K*_a_ in the presence of Ca^2+^ towards a higher value (Supplementary Fig. 2a and Supplementary Table 2).

### Imaging of new Y-GECO variants in cultured cells

**Figure 2:**
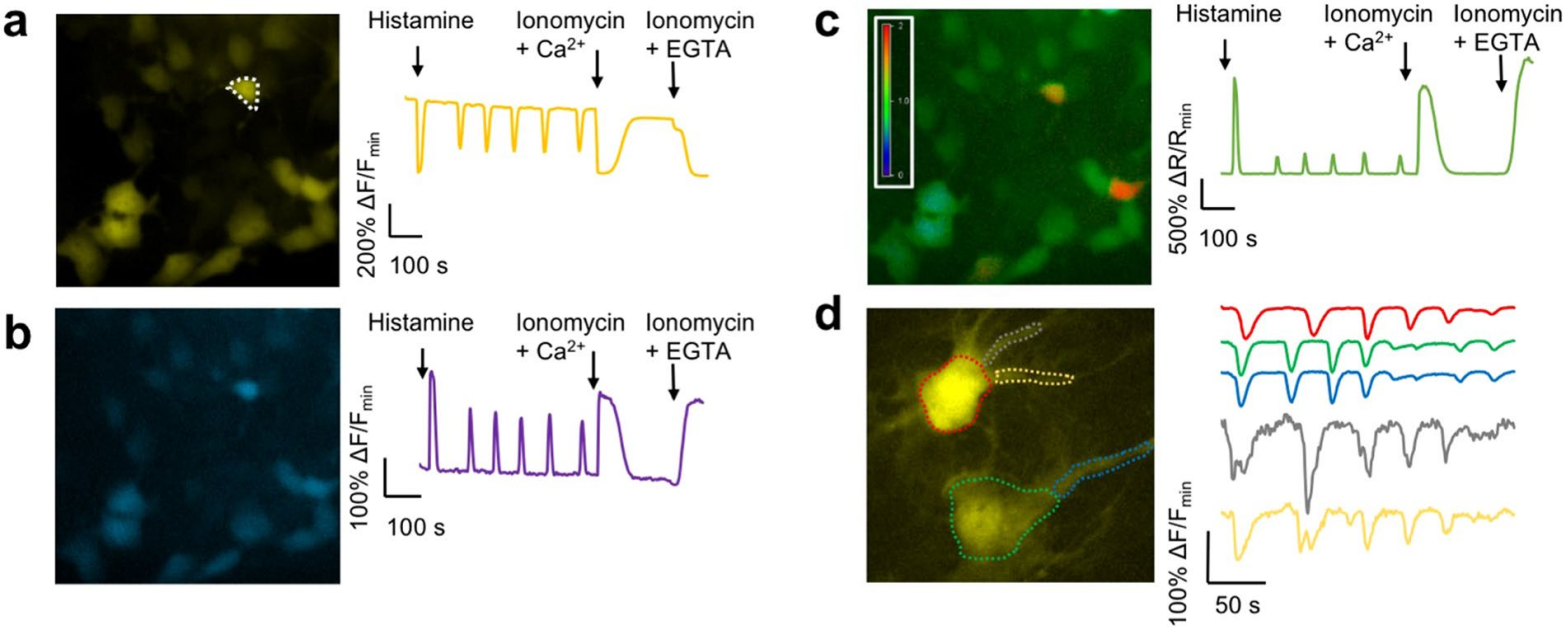
Imaging of new Y-GECO2m. (**a**–**c**) Fluorescence images of HeLa cells expressing Y-GECO2m. For each panel, the right hand chart shows fluorescence signals for the cell enclosed with a dashed-line in response to histamine-induced Ca^2+^ oscillations. (**a**) Fluorescence with excitation at 480 nm. (**b**) Fluorescence with excitation at 440 nm. (**c**) Ratiometric response (excitation at 440 nm/excitation at 480 nm). (**d**) Fluorescent image of glial cells in dissociated rat hippocampal culture expressing Y-GECO2m. The fluorescence responses of selected regions to the spontaneous Ca^2+^ changes over time are demonstrated in the traces at the right side in the same color (excitation at 480 nm). Quantitative measurements of fluorescence responses in HeLa cells is provided in Supplementary Table 4.

To demonstrate the performance of the most promising of the new Y-GECO variants for live cell imaging, genes were expressed in cultured HeLa cells and dissociated rat hippocampal cells, and fluorescence was imaged using wide-field illumination. With their near optimal *K*_d_′s for detection of cytosolic Ca^2+^ concentration changes, and reasonable dissociation kinetics, Y-GECO1m (*K*_d_′ = 190 nM) and Y-GECO2m (*K*_d_′ = 204 nM) are promising indicators for imaging of Ca^2+^ dynamics in cultured cells. Expression in HeLa cells and treatment with histamine resulted in large oscillations in fluorescence intensity and excitation ratio. To determine the maximal changes, cells were treated with EGTA/ionomycin to deplete intracellular Ca^2+^ and then Ca^2+^/ionomycin to saturate the indicator. These treatments resulted in intensity changes of 10-fold for Y-GECO2m and 6-fold for Y-GECO1m (Fig. 2a and Supplementary Table 4). Ratiometric imaging with alternating 438 nm and 480 nm excitation revealed that Y-GECO2m gives ratiometric changes approximately 2-fold greater than that of Y-GECO1m (maximal ratio changes of 35-fold for Y-GECO2m vs. 18-fold for Y-GECO1m) (Fig. 2b,c and Supplementary Table 4). Analogous experiments with Y-GECO2s, another variant with an appropriate affinity (*K*_d_′ = 121 nM) for imaging Ca^2+^ dynamics in cultured cells, revealed average intensity and ratio changes similar to those of Y-GECO2m (Supplementary Table 4). The intensiometric response of Y-GECO2m (10-fold) and Y-GECO2s (11-fold), when treated with EGTA/ionomycin followed by Ca^2+^/ionomycin, is smaller than that of the most widely used Ca^2+^ indicator GCaMP6s^9^, which we have previously determined to have a 20-fold change under similar conditions^11^. However, the ratiometric response of these Y-GECO variants does provide signal changes that are comparable to the intensiometric changes of GCaMP6s. Y-GECO2m also proved effective for imaging of slow Ca^2+^ waves when expressed in glial cells in dissociated rat hippocampal cultures (Fig. 2d).

### Imaging of neuronal inhibition in *Drosophila* using the YGECO2s series

**Figure 3:**
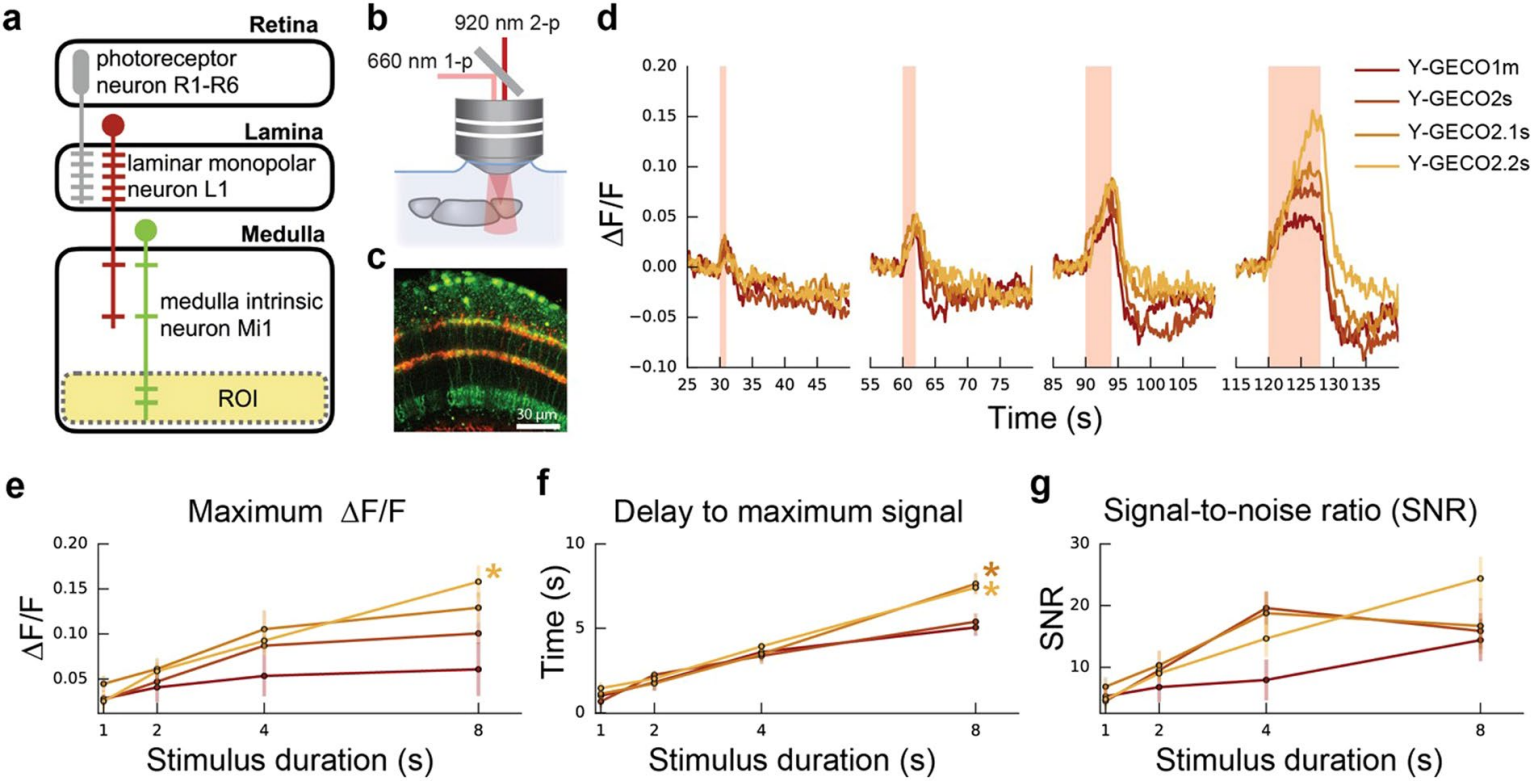
Imaging of Mi1 activation upon optogenetic activation of L1. (**a**) Schematic (modified from Strother *et al*.)^18^ featuring an L1 neuron (red) projecting from lamina to the medulla where it connects to an Mi1 neuron in layer 4. The Y-GECO response in Mi1 is measured in a region-of-interest (ROI) (faded yellow) spanning layers 8–10 (Supplementary Fig. 4b). (**b**) Y-GECO fluorescence was imaged using 2-photon excitation at 920 nm, while optogenetic activation of Chrimson was achieved using 1-photon excitation at 660 nm. (**c**) Image of L1 neurons labeled with Chrimson-tdTomato (red) and Mi neurons expressing Y-GECO1m (green). (**d**) Red light triggered Chrimson activation in L1 over durations spanning 1, 2, 4, and 8 seconds and presented here in the order they occurred during the protocol (Supplementary Fig. 4c). The median ∆F/F response for each time period is shown. Raw data is provided in Supplementary Fig. 5. (**e**,**f**) The response properties to the 1, 2, 4, and 8 second stimulations for the four Y-GECO variants are summarized. (**e**) The maximum ∆F/F reveals a trend for higher ∆F/F in inhibitory responses for indicators with a lower *K*_d_. Kruskal-Wallis H-test found a significant difference (*P* = 0.0097) among the variants only over the 8 s time period. Post-hoc analysis using paired Wilcoxon ranksum between all variant combinations only found a significant difference between Y-GECO1m and Y-GECO2.2s (*P* = 0.0278, as indicated by *). (**f**) The delay to maximum signal. Kruskal-Wallis H-test found significant difference (*P* = 0.0033) among the variants only over the 8 s time period. Post-hoc analysis using paired Wilcoxon ranksum between all variant combinations only found significant differences between Y-GECO1m and Y-GECO2.1s (*P* = 0.0404) and Y-GECO1m vs Y-GECO2.2s (*P* = 0.0063). (**g**) The signal-to-noise ratio is the maximum ΔF/F signal divided by the baseline standard deviation (5 s period before simulation started). Lines for Y-GECO variants are colored as in (**d**). Numbers tested are: Y-GECO1m = 5, Y-GECO2s = 9, Y-GECO2.1 s = 8, and Y-GECO2.2s = 9.

Since all Y-GECO variants exhibit an inverted response to Ca^2+^, we reasoned that they would be useful as positive indicator for detecting inhibitory (hyperpolarizing) responses, possibly enabling transient decreases in Ca^2+^ to be imaged with improved sensitivity. This application is similar to one reported by Hara-Kuge *et al*., who recently applied the IP2.0 inverse response indicator to visualize neuronal in the AWC^ON^ neurons of *Caenorhabditis elegans*^14^. To explore this possibility and to examine the dependence of the response on indicator *K*_d_′, we expressed a series of Y-GECO variants (specifically, those with the highest Ca^2+^-binding affinities) in the *Drosophila melanogaster* Mi1 neuron. It has previously been demonstrated that the visual system of *Drosophila* is split into two pathways: the L1 “ON” pathway for light increment, and the L2 “OFF” pathway for light decrement^16^. L1 and L2 cells in the lamina serve as primary synaptic targets of photoreceptors and send input signals to neurons in medulla such as Mi1 (for L1) and Tm1 (for L2). The Mi1 neuron acts in the ON circuit, depolarizing when light increases and hyperpolarizing when light decreases^17,18^. Here, we mimic the light decrease by optogenetically stimulating the L1 neuron that makes an inhibitory synaptic connection to Mi1^19^ (Fig. 3a). When inhibited, the free Ca^2+^ levels in Mi1 drop, a response thus far observed with Ca^2+^ indicators that increase fluorescence in response to increases in Ca^2+^ concentration^19^.

We used the Gal4/UAS system to express Chrimson^20^ in L1 neurons and the LexA/LexAOP system to express Y-GECO1m (*K*_d_′ = 190 nM), Y-GECO2s (*K*_d_′ = 120 nM), Y-GECO2.1 s (*K*_d_′ = 63 nM), or Y-GECO2.2s (*K*_d_′ = 25 nM) in Mi1 neurons (Supplementary Fig. 4a). We chose to use Y-GECO1m, rather than Y-GECO2m, due to its higher Ca^2+^ affinity and faster *k*_*off*_. Chrimson activation with red light stimulation (660 nm, 1-photon) occurred every 30 s at a constant intensity of 0.24 mW/mm^2^ with duration increasing from 1, to 2, to 4, to 8 s, followed by a final 1 s pulse (Supplementary Fig. 4c). During activation, Y-GECO fluorescence was imaged using 2-photon excitation (920 nm) in *ex vivo* preparations, in an ROI spanning medulla layers 8–10 where the dendritic arbors from Mi1 provide the largest grouped area (Fig. 3a–c and Supplementary Fig. 4b).

These experiments revealed that fluorescence response (∆F/F) of a particular Y-GECO variant corresponded with the variant’s *K*_d_′ and the length of the stimulation period (Fig. 3d,e). The correspondence between *K*_d_′ and fluorescence response is best observed during the 8 s stimulation period. For this stimulation period, the highest affinity indicator, Y-GECO2.2s, exhibited the greatest ∆F/F, followed by Y-GECO2.1s, then Y-GECO2s, and finally Y-GECO1m. All variants exhibited an increasing delay to maximum signal with increasing stimulation period (Fig. 3f). Y-GECO2.2s exhibited the greatest signal to noise ratio (SNR) for the 8 s stimulation period but the two variants with intermediate Ca^2+^ affinity, Y-GECO2s and Y-GECO2.1 s, exhibited higher SNR at the 4 s stimulation, and similar SNR at 2 s and 1 s stimulation (Fig. 3g).

An effort was made to further characterize Y-GECO variants *in vivo* in *Drosophila* using visual stimulation. In this experiment, the cuticle was removed from the back of the head to allow the Mi1 neurons to be imaged. We placed a blue light LED in front of the fly eye and used 2-photon excitation (920 nm) to image fluorescence from GCaMP6s and Y-GECO-series Ca^2+^ indicators in Mi1 during stimulus periods when the light intensity was ramped down to give the maximum response. Using this approach, we were consistently able to identify responsive neurons when we used GCaMP6s but not when we used Y-GECO variants.

## Discussion

### New Y-GECO variants with a broad range of Ca^2+^ affinities

The Y-GECO series now contains a total of seven variants with *K*_d_′ values ranging over 2 orders of magnitude. From highest to lowest affinity, this series includes: Y-GECO2.2s (*K*_d_′ = 25 nM); Y-GECO2.1 s (*K*_d_′ = 63 nM); Y-GECO2s (*K*_d_′ = 121 nM); Y-GECO1m (*K*_d_′ = 190 nM); Y-GECO2m (*K*_d_′ = 204 nM); Y-GECO2f (*K*_d_′ = 2200 nM); and Y-GECO1f (*K*_d_′ = 2500 nM). For monitoring of neural activity with a cytosolic indicator, Ca^2+^ *K*_d_′ values in the 100 to 200 nM range have been empirically found to be close to ideal, as demonstrated by the GCaMP series of highly optimized indicators^9^. Due to the fundamental relationship *K*_d_ = *k*_off_/*k*_on_, faster Ca^2+^ dissociation kinetics (described by rate constant *k*_off_) must be associated with a higher *K*_d_, assuming no changes in Ca^2+^ association kinetics (described by rate constant *k*_on_). Accordingly, all genetically encoded Ca^2+^ indicators for neural activity imaging represent compromises between *K*_d_′ (lower is better) and *k*_off_ (higher is better). As the Y-GECO series of indicators all share very similar spectral properties, they provide researchers with the opportunity to empirically test and identify the particular indicator that is best tuned to the respond to intracellular Ca^2+^ dynamics under investigation. In addition, their inverse response behavior means that decreases in Ca^2+^ concentration associated with hyperpolarization will be reported as increasing fluorescence signals, which are generally preferred for tissue imaging. Hyperpolarization causes a decrease in Ca^2+^ only if voltage-gated channels (e.g., T-type Ca^2+^ channels) are partially active at resting potential and can be further inactivated by hyperpolarization.

### Inverse response Ca^2+^ indicators for visualization of hyperpolarization

*In vivo* electrophysiological recordings^17^ and imaging with a genetically encoded voltage indicator^19^, have been used to probe depolarization of the L1 neuron of the *Drosophila* visual pathway, as induced by a light to dark transition^16^. In response to L1 depolarization, the Mi1 neuron hyperpolarizes, clearly demonstrating an inhibitory contact between L1 and Mi1. *In vivo* Ca^2+^ imaging with GCaMP6f^9^ has revealed that the intracellular Ca^2+^ concentration follows the membrane polarization (i.e., a Ca^2+^ decrease below resting levels during hyperpolarization), in layer M10 of stimulated Mi1 neurons^19^.

To further probe Ca^2+^ signaling in the Mi1 neuron, we used a series of Y-GECO indicators with *K*_d_′ values ranging from 25 nM to 190 nM and expressed them in Mi neurons at the same concentration and activated the neurons identically. These experiments revealed that the *K*_d_′ of the indicator has a substantial effect on the ΔF/F and SNR, and a lesser effect on the delay to reach the maximum signal. For an 8 s stimulation, the relationship between *K*_d_′ and ΔF/F was clear: a lower *K*_d_′ gave a higher ΔF/F. We speculate that, for the 8 s stimulation, free Ca^2+^ levels drop below *K*_d_′ (i.e., well below 25 nM) for all variants. Variants with a greater *K*_d_′ (i.e., 100–200 nM) have a reduced change in fluorescence because their *K*_d_′ is closer to the resting Ca^2+^ concentration and so they have higher fluorescence prior to stimulation and diminished ΔF/F. Consistent with their larger values of *k*_off_ (i.e., faster dissociation), the two variants with higher *K*_d_′ (Y-GECO1m and Y-GECO2s) exhibited a decreased delay to maximum fluorescence relative to the two variants with lower *K*_d_′ (Y-GECO2.1s and Y-GECO2.2s) with an 8 s stimulus. Lastly, the signal-to-noise ratio (SNR) depends on the amount of fluorescence signal acquired, which is necessarily dependent on both *K*_d_′ and *k*_off_. Accordingly, neither the highest (Y-GECO2.2s) nor the lowest (Y-GECO1m) affinity variants (slowest and fastest, respectively) gave the highest SNR at stimulus durations up to 4 s. Rather, the two middle affinity variants (Y-GECO2s and Y-GECO2.1s), which must represent appropriate compromises of affinity and off-rate kinetics, tended to provide the best SNR at stimulus durations up to 4 s. Following a stimulus duration of 8 s, the slow kinetics of the high affinity Y-GECO2.2s variant are presumably no longer limiting and so this variant provides the highest SNR within the series.

While we succeeded at using Y-GECO variants to image neuronal inhibition in an *ex vivo* tissue preparation, we were unable to detect responses from Y-GECO with natural light stimulation *in vivo*. We suspect that a poor 2-photon cross section for Y-GECO variants is the primary reason for this discrepancy. For the *in vivo* experiments, it was challenging to discern Y-GECO expressing cells from background using 2-photon excitation. Increasing the laser power led to photobleaching which further decreased the SNR. Although it is beyond the scope of this work, we suggest that using 1-photon excitation would provide improved performance, though this would be accompanied with decreased penetration of light into the tissue. Further optimization of the Y-GECO 2-photon cross section and photostability could enhance its application for *in vivo* imaging.

### Summary

Despite the fact that 30% of neurons in humans and *Drosophila* are inhibitory, there are relatively few optogenetic indicators optimized for imaging of inhibitory neuronal activity. We, and others^14^, propose that, for imaging of inhibitory activity, inverse response Ca^2+^ indicators have an inherent advantage to direct response Ca^2+^ indicators. Specifically, imaging of inhibitory activity with a direct response Ca^2+^ requires detection of a dimming response that could be readily obscured by out of focus fluorescence from adjacent bright cells. In contrast, inhibitory activity could be more easily detected with an inverse response Ca^2+^ indicator due to the diminished background fluorescence originating from adjacent out-of-focus cells.

Our results suggest that, for an inverse response indicator in the Mi1 neuron, a *K*_d_′ of less than 100 nM produces greater changes in fluorescence without compromising the response time. In contrast, direct response Ca^2+^ indicators optimized for detection of neuronal action potentials range have been found to perform best when the *K*_d_′ is tuned to the 100 to 200 nM range. An important caveat is that this conclusion applies only to the Mi1 neuron within the synaptic region studied. Other neurons or even regions within the same neuron could have different resting Ca^2+^ levels which would change the optimum *K*_d_′. For this reason, we advocate the empirical identification of the optimal *K*_d_′ by testing a series of variants such as the ones described in this work.

## Methods

### Site-directed mutagenesis for mutation introduction

Mutations were introduced by Quikchange II Site-directed Mutagenesis kit (Agilent Technologies) with primers containing desired mutations at specific positions.

### Purification and *in vitro* characterization of Y-GECO proteins

The procedure for purification and determination of extinction coefficient, quantum yield, and *K*_d_′ of Y-GECO variants has been previously described^11^. A DU-800 UV-vis spectrophotometer (Beckman) was used to measure absorption spectra, and a Safire2 plate reader (Tecan) was used to measure the excitation and emission spectra. The ratiometric response to Ca^2+^ of Y-GECO is defined as (R_max_ − R_min_)/R_min_, where R = (I with 526 nm excitation)/(I with 416 nm excitation), and I is the fluorescence intensity at 550 nm. The intensiometric response to Ca^2+^ of Y-GECO is defined as (I_max_ − I_min_)/I_min_, where I is the fluorescence intensity at 550 nm with 526 nm excitation.

### Kinetics of Ca^2+^ association and dissociation fluorescence change of Y-GECO

Stopped-flow spectroscopy was used to evaluate reaction kinetics of FP with Ca^2+^, using an excitation wavelength of 520 nm, a 13.95 nm bandwidth, and an emission wavelength of 540 nm with a 37 nm bandwidth. *E*. *coli* cells expressing Y-GECO variants were first suspended in TBS buffer containing 0.2 mM CaCl_2_ or 0.2 mM EGTA, followed by rapid mixing (1:1) with TBS buffer containing 20 mM EGTA or 20 mM CaCl_2_. Fluorescence signals were captured using ProData SX software.

Ca^2+^-dissociation kinetics of purified Y-GECO indicators were measured as described previously^11^. The fluorescence intensity vs. time was collected, plotted and fit to a single exponential curve, giving k_off_.

### Construction of plasmids for mammalian cell expression

To express Y-GECO indicators in HeLa cells and dissociated hippocampal cultures for *ex vivo* characterizations, the Y-GECO gene in pBAD vector, used for expression in *E*. *coli* cells, was amplified by PCR with the primers FW_BamHI_Kozak_6His and RV_CaM_stop_EcoRI, followed by gel purification of the PCR products. The purified gene was then digested with the restriction enzymes BamHI and EcoRI, purified and ligated into a modified pcDNA3 plasmid vectors which have been digested with the same enzymes and purified by gel. The ligation products, the plasmids for mammalian expression of Y-GECO indicators, were transformed into electrocompetent *E*. *coli* DH10B cells which were then plated on an agar plate containing 1× ampicillin for overnight culture at 37 °C. On the following day, individual colonies were picked for 12 h liquid culture in 4 mL B/ampicillin, shaken at 250 rpm. The cultured cells were then isolated and the plasmids were purified for mammalian cell transfection and expression.

### HeLa cell culture and imaging

HeLa cells were cultured and transfected as described previously^11^. Wide-field imaging of cells was performed on an epifluorescence inverted microscope (Eclipse Ti-E, Nikon) equipped with a digital CCD camera (QuantEM 512SC). The microscope and camera were controlled using NIS-Elements Advanced Research software. Cells were imaged with a 20× air objective lens (NA 0.8). The cells were illuminated by a 100 W mercury arc lamp and a 25% neutral density filter was used to reduce the intensity of the light. To record the long Stokes shift fluorescence, we used a filter set of 438/24 nm (excitation), 458 nm (dichroic) and 542/27 nm (emission). Exposure time was set to 700 ms. To record the short Stokes shift fluorescence, we used a filter set of 480/40 nm (excitation), 505 (dichroic) nm and 535/40 nm (emission), with exposure time 500 ms.

To image histamine induced Ca^2+^ dynamics, images were acquired every 4 s for ~20 min. After ~30 s of initial recording, 100 μM histamine solution was added to the dish to reach a final concentration of 10 μM. After ~10 min recording, 10 mM EGTA, 40 μM ionomycin in Ca^2+^-and Mg^2+^-free HHBSS was then added to reach a final concentration of 1 mM EGTA, 4 μM ionomycin. Then, 20 mM Ca^2+^, 40 μM ionomycin in Ca^2+^ and Mg^2+^ free HHBSS was then added to reach a final concentration of 2 mM Ca^2+^, 4 μM ionomycin.

### Dissociated rat hippocampal culture preparation and imaging

Dissociated rat hippocampal cells are prepared and transfected as described previously^11^. Tissues were collected from animals that were being sacrificed for unrelated experiments that were approved by the University of Alberta Animal Care and Use Committee and carried out in compliance with guidelines of the Canadian Council for Animal Care and the Society for Neuroscience’s Policies on the Use of Animals and Humans in Neuroscience Research. Y-GECO indicators were imaged under conditions similar to those described above. A 60× oil objective lens was used for higher amplification and a 12.5% neutral density filter was used to reduce the excitation intensity. Only short-Stokes fluorescence was recorded by using the filter set 480/40 nm (excitation), 505 (dichroic) nm and 535/40 nm (emission). Exposure time was 69 ms and images were acquired every 1 s for 4 min.

### *Drosophila* imaging

Y-GECO variants were codon optimized for *Drosophila*, cloned into 13XLexAOP2-IVS-Syn21-[insert]-p10 plasmid (gift from Barret Pfeiffer) and inserted into the genome at the su(Hw)attP8 landing site. Brains from females (Genotype: 10xUAS-Chrimson-tdTomato (attP18)/LexAOP2-Y-GECO (suHwattP8); 19F01-LexA (suHwattP5) (Mi1)/+; 27G06-GAL4 (attP2) (L1)/+) expressing Chrimson-tdTomato in L1 neurons and Y-GECO variants in Mi1 neurons were tested.

Flies were reared at 25 °C on retinal supplemented (0.2 mM) cornmeal medium that was shielded from light. All experiments were performed on female flies, 1–4 days after eclosion. Brains were dissected in a saline bath (103 mM NaCl, 3 mM KCl, 2 mM CaCl_2_, 4 mM MgCl_2_, 26 mM NaHCO_3_, 1 mM NaH_2_PO_4_, 8 mM trehalose, 10 mM glucose, 5 mM TES, bubbled with 95% O_2_/5% CO_2_). After dissection, the brain was positioned anterior side up on a coverslip in a Sylgard dish submerged in 3 ml saline at 20 °C.

The sample was imaged with a resonant scanning 2-photon microscope with near-infrared excitation (920 nm, Spectra-Physics, INSIGHT DS DUAL) and a 25× objective (Nikon MRD77225 25XW). The microscope was controlled by using ScanImage 2015.v3 (Vidrio Technologies)^21^. Images were acquired with 141 μm × 141 μm field of view at 512 × 512 pixel resolution, approximately 9 Hz frame rate after averaging 5 frames. The excitation power for Ca^2+^ imaging measurement was 12 mW.

For the photostimulation, the light-gated ion channel Chrimson was activated with a 660 nm LED (M660L3 Thorlabs) coupled to a digital micromirror device (Texas Instruments DLPC300 Light Crafter) and combined with the imaging path with a FF757-DiO1 dichroic (Semrock). On the emission side, the primary dichroic was Di02-R635 (Semrock), the detection arm dichroic was 565DCXR (Chroma), and the emission filters were FF03-525/50 and FF01-625/90 (Semrock). Photostimulation light was delivered in a pulse train that consisted of three 5 pulses with increasing pulse durations (1, 2, 4, 8 and 1 seconds) every 30 seconds as outlined in Supplementary Fig. 4c. The light intensity was 0.24 mW/mm^2^, as measured using Thorlabs S170C power sensor.

In custom python scripts, an ROI was drawn over layers M8–10 on a figure containing the standard deviation over time. Before calculating the change in fluorescence (ΔF), the offset was subtracted from the fluorescence and then baseline fluorescence was subtracted. Baseline fluorescence is the median fluorescence over a 5 s time period before stimulation started. The ΔF was then divided by baseline to normalize signal (ΔF/F). The final signal was run through a gaussian filter (sigma = 1).

The time period included in the maximum fluorescence, delay to maximum signal, and signal to noise ratio start from stimulation start to 2 s after stimulation ended. Signal to noise ratio was calculated by taking the maximum ΔF/F signal and dividing by the baseline (5 s period before simulation started) standard deviation.

### Data availability

The datasets generated during and/or analyzed during the current study are available from the corresponding author on reasonable request.

## Electronic supplementary material

Supplementary Information
