# Supplementary material for: Inverse-response Ca2+ indicators for optogenetic visualization of neuronal inhibition
Source: Sci Rep. 2018 Aug 6;8:11758. doi: 10.1038/s41598-018-30080-x (PMC6079023; doi:10.1038/s41598-018-30080-x)
Supplement: Supplementary file 1 — Supplementary Information [file 41598_2018_30080_MOESM1_ESM.docx]

**Supplementary Material**

**Inverse-response Ca^2+^ indicators for optogenetic visualization of neuronal inhibition**

Yufeng Zhao^1^, Daniel Bushey^2^, Yongxin Zhao^1,3^, Eric R. Schreiter^2^, D. Jed Harrison^1^, Allan M. Wong^2^*, and Robert E. Campbell^1^*

^1^ Department of Chemistry, University of Alberta, Edmonton, AB T6G 2G2, Canada.

^2^ Howard Hughes Medical Institute, Janelia Research Campus, Ashburn, VA 20147, USA.

^3^ Present address: Department of Biological Sciences, Carnegie Mellon University, Pittsburgh, PA 15213, USA.

**Supplementary Figures**


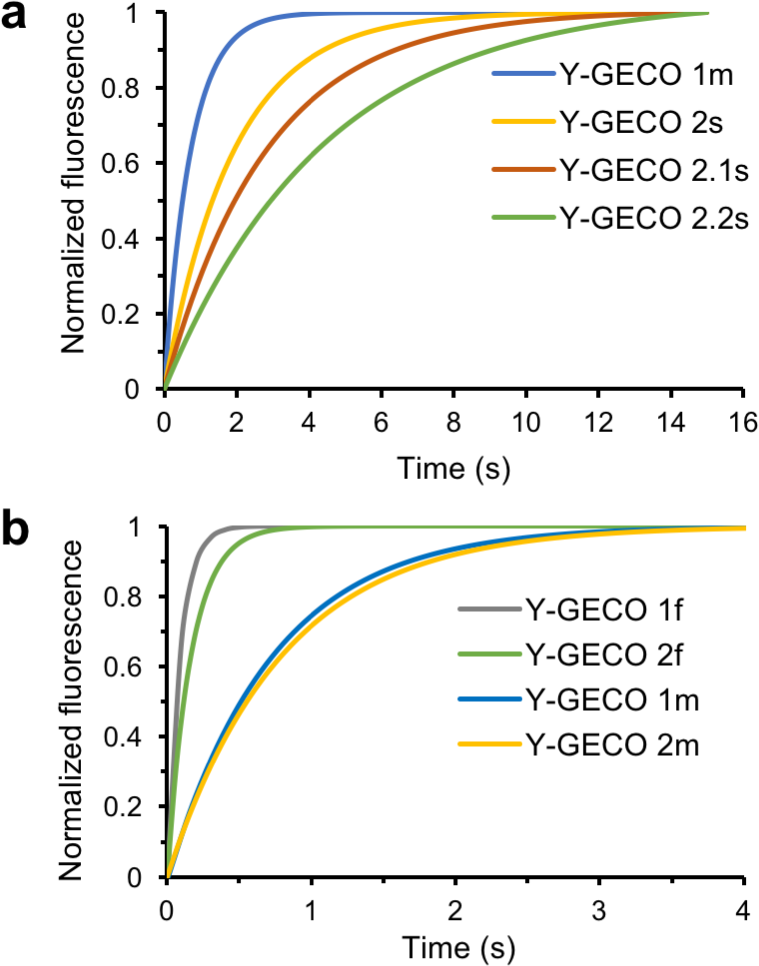


**Supplementary Figure 1. Ca^2+^ dissociation kinetics for Y-GECO variants.** (**a**) Ca^2+^-dissociation kinetics of Y-GECO1m, 2s, 2.1s, and 2.2s**. (b)** Ca^2+^-dissociation kinetics of Y-GECO1f, 2f, 1m (reproduced from (**a**) for the sake of reference), and 2m. Y-GECO1f and 2f data from Ref. [16](https://paperpile.com/c/HXIFXx/lRgy).

**
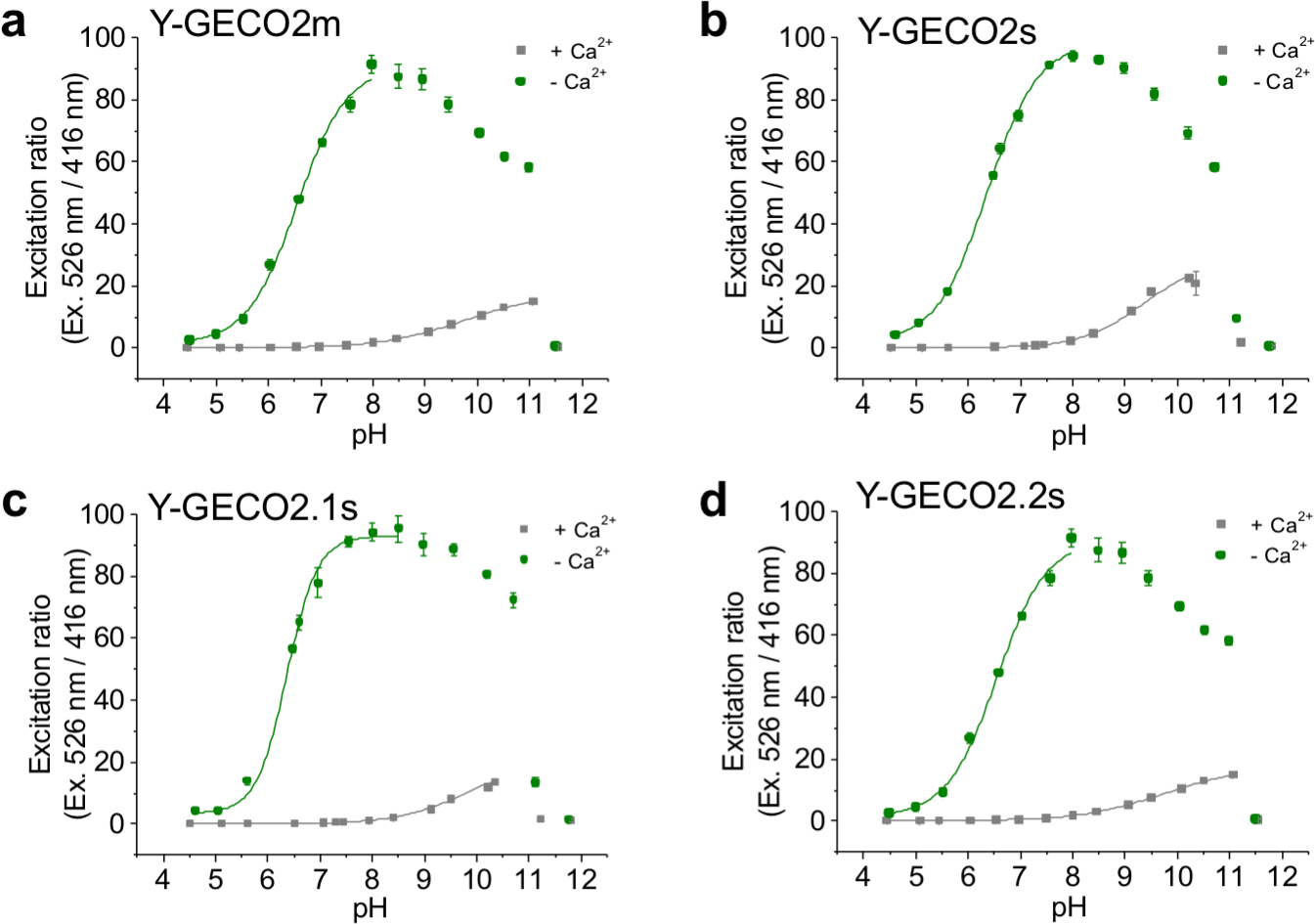
Supplementary Figure 2. pH titrations of Y-GECO variants.** (**a**) Y-GECO2m. (**b**) Y-GECO2s. (**c**) Y-GECO2.1s. (**d**) Y-GECO2.2s.


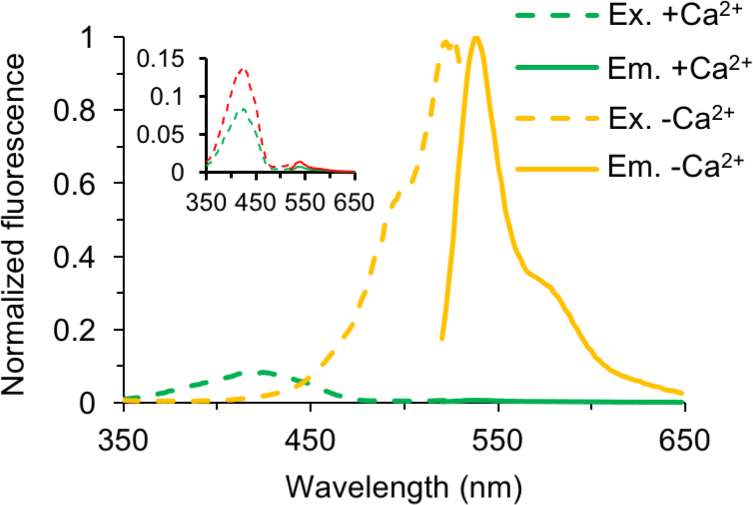


**Supplementary Figure 3. Fluorescence spectra for Y-GECO2m in the presence and absence of Ca^2+^.** Excitation (dashed lines) and emission (solid lines) spectra in the Ca^2+^-free (yellow) and Ca^2+^-bound states (green). Inset is the excitation (dashed lines) and emission (solid lines) spectra of Y-GECO2m (green) and Y-GECO1m (red) in Ca^2+^-bound state with zoomed-in y-axis.


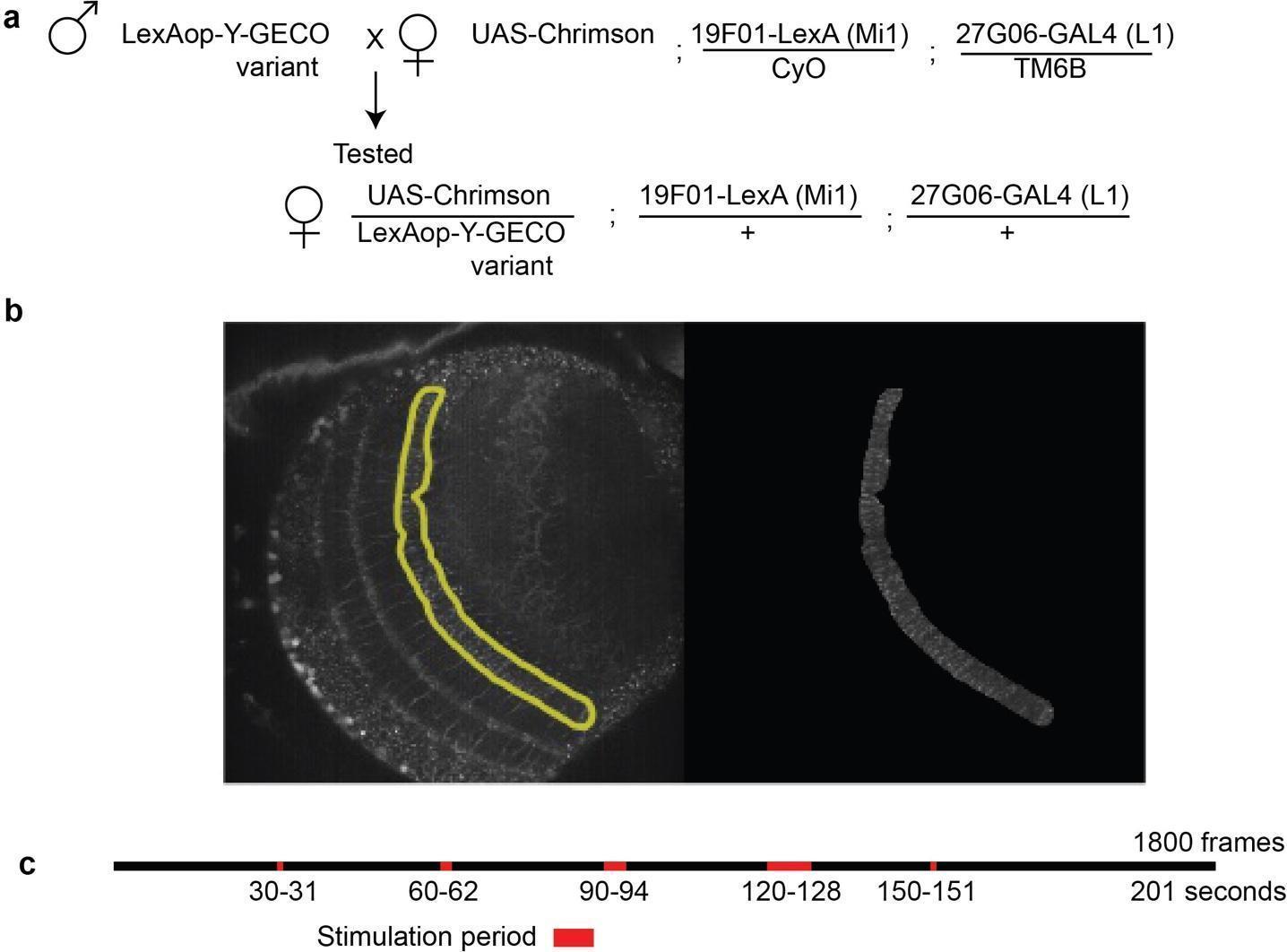


**Supplementary Figure 4. Generation of transgenic flies and imaging protocol.** (**a**) Crossing scheme used to generate females expressing the Y-GECO variants. The GAL4/UAS system was used to drive expression of Chrimson in L1 neurons, and the LexA/LexAop system was used to drive expression in Mi1 neurons. Males passed a LexAop driven Y-GECO while females passed the UAS-Chrimson (10×UAS-Chrimson-tdTomato) driver lines 19F01-LexA and 27G06-GAL4. The 27G06-GAL4 drives Chrimson expression in the L1 neurons while the 19F01-LexA drives Y-GECO variant expression in the Mi1 neurons. (**b**) Representative image (left) shows the standard deviation in the medulla over the time series from a single plane from a Y-GECO2.2s fly. Outline in yellow is the ROI used to measure changes in fluorescence in the M8-10 layer. Image (right) shows the standard deviation after application of a mask defined by the ROI at left. (**c**) Stimulation protocol where red light is turned on (represented in red) at a constant intensity and durations of 1 s, 2 s, 4 s, 8 s and back to 1 s at 30 s intervals.


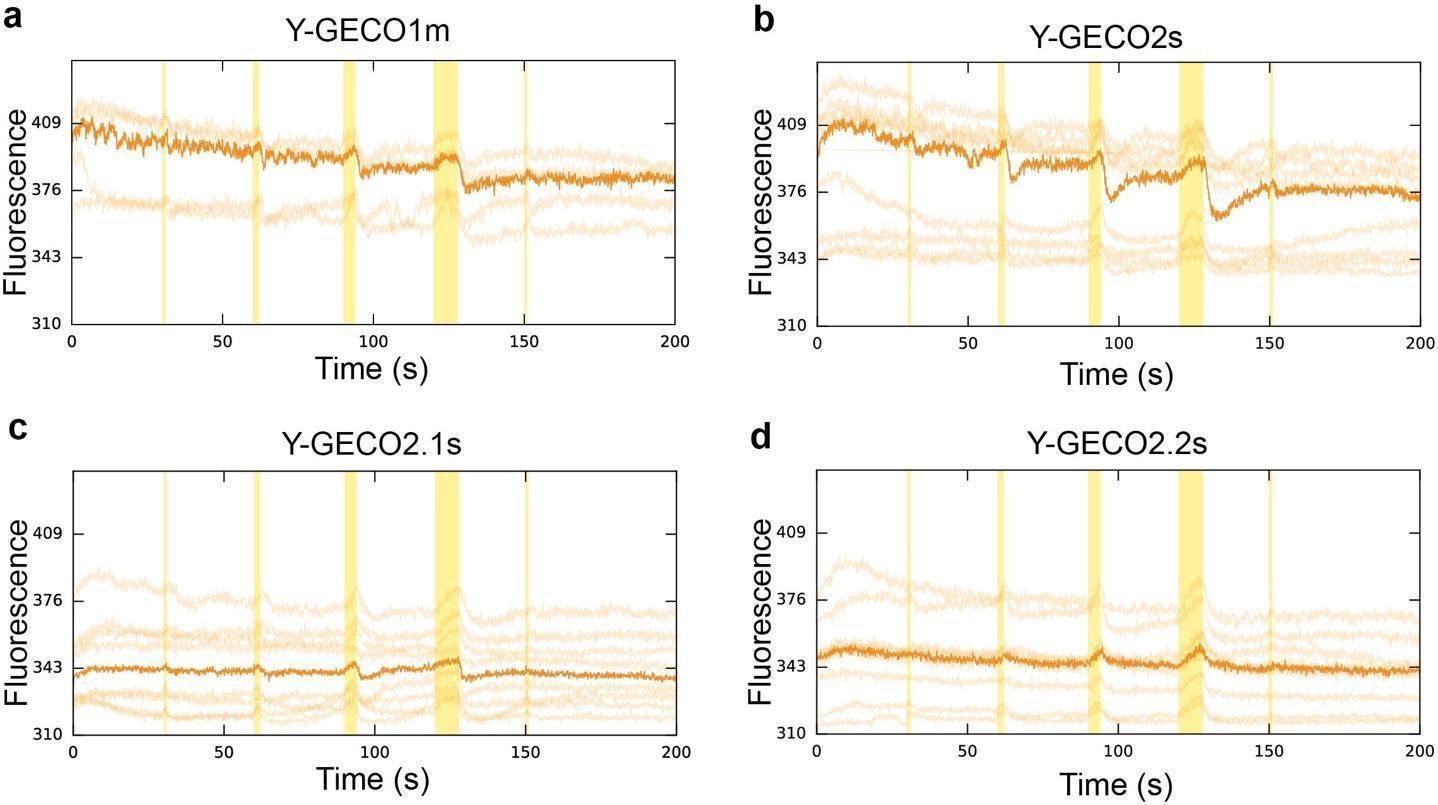


**Supplementary Figure 5. Raw fluorescence traces from fly imaging.** Raw fluorescence measured from the Y-GECO variants tested over the time series. Shaded orange represents individual trials. Dark orange is the median result.

**Supplementary Tables**

**Supplementary Table 1. Mutations in Y-GECO variants.**

| Variant | Mutations |
| --- | --- |
| Y-GECO1m | - |
| Y-GECO1f | ΔG13^a^ |
| Y-GECO2f^b^ | ΔG13, M1T, T30S, S128P |
| Y-GECO2m | ΔG13, M1T, T30S, S128P, Q276D, M300I, L309F |
| Y-GECO2s | M300I |
| Y-GECO2.1s | Q276D, M300I |
| Y-GECO2.2s | Q276D, M300I, L309F |

^a^ ΔG13 represents that G13 is deleted.

^b^ Data from Ref. 16.

**Supplementary Table 2. Properties of Y-GECO variants.**

| Protein variant | Ca^2+^ | λ_abs_ (nm) with ε (mM^-1^cm^-1^) in parentheses | ϕ ^a^ with λ_ex_, λ_em_ (nm) in parentheses | ε*ϕ ^b^ | Ca^2+^-dependent change in brightness^c^ with λ_ex_ (nm) in parentheses | p*K*_a_^d^ |
| --- | --- | --- | --- | --- | --- | --- |
| Y-GECO1f^e^ | - | 412 (17)  522 (26) | 0.05 (412, 538) 0.75 (522, 538) | 0.95  19.5 | ↑ 4× (412)  ↓ 6× (522) | 6.3 |
|  | + | 412 (23)  522 (6) | 0.16 (412, 538) 0.58 (522, 538) | 3.7  3.5 |  | 8.5 |
| Y-GECO1m | - | 412 (18)  522 (25) | 0.09 (412, 538)  0.85 (522, 538) | 1.6  21.3 | ↑ 6× (412)  ↓ 19× (522) | 6.5 |
|  | + | 412 (26)  522 (2) | 0.35 (412, 538)  0.56 (522, 538) | 9.1  1.1 |  | 8.8 |
| Y-GECO2f^e^ | - | 412 (20)  522 (31) | 0.06 (412, 538)  0.77 (522 538) | 1.2  23.9 | ↑ 3× (412)  ↓ 24× (522) | 6.4 |
|  | + | 412 (30)  522 (2) | 0.09 (412, 538)  0.51 (522, 538) | 2.7  1.0 |  | 9.1 |
| Y-GECO2m | - | 412 (20)  522 (26) | 0.08 (412, 538)  0.83 (522, 538) | 1.6  21.6 | ↑ 3× (412)  ↓ 54× (522) | 6.5 |
|  | + | 412 (29)  522 (1) | 0.16 (412, 538)  0.40 (522, 538) | 4.6  0.4 |  | 9.5 |
| Y-GECO2s | - | 412 (17)  522 (25) | 0.06 (412, 538)  0.74 (522, 538) | 0.7  18.5 | ↑ 14× (412)  ↓ 26× (522) | 6.3 |
|  | + | 412 (25)  522 (2) | 0.39 (412, 538)  0.33 (522, 538) | 9.8  0.7 |  | 8.9 |
| Y-GECO2.1s | - | 412 (18)  522 (25) | 0.05 (412, 538)  0.71 (522, 538) | 0.8  17.8 | ↑ 13× (412)  ↓ 59× (522) | 6.3 |
|  | + | 412 (26)  522 (1) | 0.39 (412, 538)  0.32 (522, 538) | 10.1  0.3 |  | 9.3 |
| Y-GECO2.2s | - | 412 (17)  522 (23) | 0.05 (412, 538)  0.71 (522, 538) | 0.9  16.3 | ↑ 13× (412)  ↓ 54× (522) | 6.5 |
|  | + | 412 (27)  522 (1) | 0.44 (412, 538)  0.33 (522, 538) | 11.9  0.3 |  | 9.6 |

^a^ Quantum yield ϕ for emission at the 538 nm peak measured at the two excitation peaks.

^b^ The product of ε and ϕ is proportional to the overall fluorescent brightness in units of mM^-1^cm^-1^.

^c^ Up and down arrows indicate fluorescence increases and decreases, respectively.

^d^ The p*K*_a_ is defined as the pH at which the fluorescence intensity ratio (excitation at 526 nm / excitation at 416 nm) is 50% of maximum.

^e^ Data from Ref. 16.

**Supplementary Table 3. *In* *vitro* *k*_off_ and *K*_d_’ of Y-GECO variants**

| Protein variant | *k*_off_ (s^-1^) | *K*_d_’ (nM) with  Hill coefficient in parenthesis |
| --- | --- | --- |
| Y-GECO1f^a^ | 11.65 | 2500 (2.7) |
| Y-GECO1m | 1.41 | 190 (2.6) |
| Y-GECO2f^a^ | 5.96 | 2200 (1.4) |
| Y-GECO2m | 1.23 | 204 (2.4) |
| Y-GECO2s | 0.53 | 121 (2.2) |
| Y-GECO2.1s | 0.35 | 63 (2.3) |
| Y-GECO2.2s | 0.22 | 25 (2.5) |

^a^ Data from Ref. 16.

**Supplementary Table 4. Characterization of Y-GECO variants in HeLa cells.** Cells were treated first with histamine (abb. His), then with EGTA/ionomycin (abb. EGTA), and then with Ca^2+^/ionomycin (abb. Ca^2+^).

| Protein variant | n^a^ |  | Maximum EGTA to minimum Ca^2+^ ratio^b^ | Maximum His to minimum His ratio^c^ | His to EGTA/Ca^2+^ ratio of ratios^d^ |
| --- | --- | --- | --- | --- | --- |
| Y-GECO1m | 33 | Intensity^e^ | 6 ± 4 | 1.8 ± 0.5 | 0.5 ± 0.3 |
|  |  | Ratio^f^ | 18 ± 14 | 4 ± 2 | 0.4 ± 0.4 |
| Y-GECO2m | 47 | Intensity | 10 ± 5 | 2.2 ± 0.6 | 0.3 ± 0.2 |
|  |  | Ratio | 35 ± 19 | 6 ± 3 | 0.3 ± 0.2 |
| Y-GECO2s | 48 | Intensity | 11 ± 5 | 3 ± 1 | 0.4 ± 0.2 |
|  |  | Ratio | 30 ± 16 | 8 ± 5 | 0.3 ± 0.2 |

^a^ Number of transfected HeLa cells imaged for each variant.

^b^ The highest fluorescence response after EGTA treatment to the lowest response after Ca^2+^ treatment.

^c^ The highest fluorescence response to the lowest response after His treatment.

^d^ The ratio of (max His to min His) to (max EGTA to min Ca^2+^).

^e^ Intensity with 480/40 nm excitation and 535/40 nm emission.

^f^ Ratio of intensity with 438/24 nm excitation and 542/27 nm emission divided by intensity with 480/40 nm excitation and 535/40 nm emission.

**Supplementary Table 5. Oligonucleotides used in this work**

| Name | 5’ to 3’ sequence |
| --- | --- |
| FW_XbaI_6His | GCGATGTCTAGAGGTTCTCATCATCATCATCATCATGGTATGGCTAGC |
| RV_stop_HindIII | GCGATGAAGCTTCTACTTCGCTGTCATCATTTGTACAAACTCTTCGTAGTTT |
| FW_BamHI_Kozak_6His | AAACAGGAGGAATTAAGCTTGGGATCCACCATGGGTTCTCATCATCATCATCATCATGGTATGGC |
| RV_CaM_stop_EcoRI | CGCGAATTCCTACTTCGCTGTCATCATTTGTAC |
| FW_ M300I | CGGGGATGGGACGATAACAACCGAGGAG |
| FW_ Q276D | GCTGACACGCGTGACGACCTGACTGAAGAGCAG |
| FW_ L309F | GCTGGGGACGGTGTTCCGGTCTCTGGGGC |
